# Evaluation of the Antifungal and Antiproliferative Properties of the Lichen *Roccella tinctoria* DC. Extracts and Main Components

**DOI:** 10.3390/pharmaceutics16030331

**Published:** 2024-02-27

**Authors:** Claudio Frezza, Dalia Rosa Fraioli, Francesca Conti, Roberta Maria Nicolosi, Luigi Scipione, Ilaria Serafini, Rita Petrucci, Paola Di Matteo, Daniele Rocco, Silvia Di Giacomo, Antonella Di Sotto, Graziana Bonincontro, Giovanna Simonetti, Stefania Garzoli, Daniela De Vita, Sebastiano Foddai

**Affiliations:** 1 Dipartimento di Biologia Ambientale, Università di Roma “La Sapienza”, Piazzale Aldo Moro 5, 00185 Rome, Italy; claudio.frezza@uniroma1.it (C.F.); daliarosa.fraioli@gmail.com (D.R.F.); conti.francesca1998@gmail.com (F.C.); robertamaria.nicolosi@uniroma1.it (R.M.N.); graziana.bonincontro@gmail.com (G.B.); sebastiano.foddai@uniroma1.it (S.F.); 2 Dipartimento di Chimica e Tecnologia del Farmaco, Università di Roma “La Sapienza”, Piazzale Aldo Moro 5, 00185 Rome, Italy; luigi.scipione@uniroma1.it (L.S.); stefania.garzoli@uniroma1.it (S.G.); 3 Dipartimento di Chimica, Università di Roma “La Sapienza”, Piazzale Aldo Moro 5, 00185 Rome, Italy; ilaria.serafini@uniroma1.it; 4 Dipartimento di Scienze di Base e Applicate per Ingegneria, Università di Roma “La Sapienza”, Via Castro Laurenziano 7, 00161 Rome, Italy; rita.petrucci@uniroma1.it (R.P.); p.dimatteo@uniroma1.it (P.D.M.); daniele.rocco@uniroma1.it (D.R.); 5 Dipartimento di Fisiologia e Farmacologia “V. Erspamer”, Università di Roma “La Sapienza”, Piazzale Aldo Moro 5, 00185 Rome, Italy; silvia.digiacomo@iss.it (S.D.G.); antonella.disotto@uniroma1.it (A.D.S.); 6 Dipartimento di Sicurezza Alimentare, Nutrizione e Sanità Pubblica Veterinaria, Istituto Superiore di Sanità, 00161 Roma, Italy

**Keywords:** *Roccella tinctoria* DC., different extracts, phytochemical analysis, antifungal activity, antiproliferative activity, isolated compounds

## Abstract

In this work, phytochemical analysis on different extracts of *Roccella tinctoria* DC. was reported using different techniques with respect to the past. Twenty volatile and three non-volatile compounds were identified, some of which were found in this species for the first time. The methanolic extracts and their non-volatile components were then evaluated for their antitumor effects in cancerous A549 and Mz-ChA-1 cells and for their tolerability in non-cancerous BEAS-2B and H69 cells, showing IC_50_ values from 94.6 µg/mL to 416.4 µg/mL, in general. The same extracts and compounds were also tested for their antifungal effects in *Candida albicans,* with only compound **2** being active, with an MIC_50_ value of 87 µg/mL. In addition, they were tested for their anti-*Candida* adhesion activity, anti-*Candida* biofilm formation, and anti-*Candida* mature biofilm inhibition, with efficacy percentages generally above 50% but not for all of them. Lastly, the DF3 extract and compounds **1**–**2** were tested in vivo according to the *Galleria mellonella* survival assay, showing positive mortality rates above 50% at different concentrations. All these biological assays were conducted on this species for the first time. Comparisons with other lichens and compounds were also presented and discussed.

## 1. Introduction

Lichens are a symbiotic association of a fungus defined as a mycobiont and usually represented by an ascomycete, and an alga or cyanobacterial species defined as photobiont [1], that form composite thalli, which are clearly visible, even to the naked eye [2]. They appear in a wide variety of habitats and can even survive under severe conditions (i.e., low temperatures and prolonged darkness), producing high amounts of characteristic phenolics in response to the environment [3].

*Roccella* DC. (Roccellaceae) is a genus of fruticose lichens that comprises more than 20 species, 7 of which are widespread in Europe, especially along the Mediterranean coasts [4]. Under the phytochemical point of view, about fifty secondary metabolites belonging to several classes of compounds have been reported for the *Roccella* genus, including depsides (e.g., erythrin, lecanoric acid, and lepraric acid), monoaromatic phenols (e.g., β-orcinol, ethyl orsellinate, and montagnetol), and aliphatic acids (e.g., roccellic acid) [5], which have already been reported to have strong effects against different fungal strains [6,7,8].

*Roccella tinctoria* DC. represents the most common species of this genus in Italy [9], where it is characterized by a rigid fruticose brownish thallus, 3–20 cm long smooth branches, a bark of hyphae anticlinally disposed, and, generally, the total absence of apothecia [10]. In Europe, *Roccella tinctoria* DC. is mainly known for its mass use as a source of purple dye [11]. Just a couple of works have studied its phytochemical constituents and only by means of MS, HPLC-MS, Raman spectroscopy, FT-IR, and UV, starting from differently adjusted aqueous solutions, identifying several orcein derivatives as main compounds together with the typical compounds already found in the genus [12,13,14,15]. Indeed, no phytochemical analysis has ever been conducted on different non-aqueous extracts of *R. tinctoria* collected in Italy using methods like CC and SPME.

Lichen extracts and their derived phenolic compounds have already shown promising antifungal and antiproliferative properties [16,17,18,19,20,21,22]. 

*Candida albicans* is an opportunistic fungal pathogen and a commensal yeast that colonizes the oral, gastrointestinal, and vaginal tracts [23]. Several factors, such as stress and an impaired immune system, can cause its overgrowth and mucosal and/or systemic infections [24]. *C. albicans* can make a transition from commensal to pathogen due to its virulence factors, including the ability to form biofilms [25], which represents a significant clinical problem. In fact, fungal biofilms are less susceptible to many drugs [26]. Therefore, there is a need to develop new agents active against planktonic cells and biofilms of *C. albicans*. The role of natural products in drug discovery is widely recognized in several therapeutic areas, mainly in infectious disease [27]. Several plant extracts and their metabolites have been investigated as anti-*C. albicans* agents [28,29,30]. Conversely, only a couple of studies have focused on lichen extracts [16,31] and their metabolites [32,33] but none specifically on *R. tinctoria* and its metabolites.

Several studies also investigated the in vitro antitumor activity of lichen extracts and their metabolites [22]. For instance, the methanolic extracts from *Xanthoria parietina* (L.) Th. Fr. exhibited antiproliferative properties in MDA-MB231 and MCF-7 breast cancer cells and murine myeloma cells [34], while those from *Cladonia foliacea* (Huds.) Willd. and *Hypogymnia physodes* (L.) Nyl. were in HCT-116 colon cancer cells [35]. Moreover, the methanolic extracts of *Usnea intermedia* (A. Massal.) Jatta as well as the acetone extracts from *Parmotrema gardneri* (Du Rietz) Hale, *C. foliacea* and *Parmelia arseneana* Gyelnik produced cytotoxic effects in lung cancer A549 cells [36,37,38]. Promising antiproliferative effects have also been observed for the methanolic extract from *Roccella montagnei* Bél., in human colon (DLD-1, SW-620), breast (MCF-7), head and neck (FaDu) cancer cell lines, ascribed to the presence of roccellic acid [39].

Based on this evidence, the present study aimed at characterizing the anti-*Candida* activity, the cytotoxicity in human cancer cells, and tolerability in non-cancerous ones of *R. tinctoria* extracts and their main phenolic compounds. Notably, there is a serious lack of previous investigations on these aspects.

## 2. Materials and Methods

### 2.1. Chemicals and Instruments

All solvents as well as deuterated solvents of isotopic purity were purchased from Sigma Aldrich (Milan, Italy). A Bruker AC 400 Ultrashield 10 spectrophotometer (400 MHz) (Bruker, Billerica, MA, USA) was used to record ^1^H NMR spectra at 27 °C; chemical shifts are expressed in δ (ppm), while coupling constants are in Hz. Melting points were measured on a Bobby Stuart Scientific SMP1 melting point apparatus (Bibby Scientific, Stone, UK). Infrared spectra were recorded on a PerkinElmer Spectrum-One spectrophotometer (Perkin Elmer, Shelton, CT, USA). Column chromatography on silica gel (Merck, Darmstadt, Germany; 70–230 mesh) was employed to separate the components of the extracts. Thin-layer chromatography (TLC) was performed by using aluminum-baked silica gel plates (Fluka, Honeywell, Charlotte, NC, USA; DC-Alufolien Kieselgel 60 F254). Developed plates were visualized by UV light (254 and 365 nm). Direct infusion ESI-MS/MS experiments were carried out by a Quattro Micro Tandem MS/MS with a Waters ESI source (Micromass, Manchester, UK), by direct infusion of the samples into the ESI source through an external syringe, flow rate 5 μL/min. Mass spectral data for each compound were acquired for 2 min in the appropriate mass range in both positive (ES+) and negative (ES-) ionization: cone voltage 20 V, ionization source temperature 100 °C, desolvation gas temperature of 150 °C, cone gas flow of 30 L/h, desolvation gas flow 400 L/h. Fragmentation patterns in both ES+ and ES− were obtained by using argon as collision gas and collision energy CE in a range of 10–17 eV, optimized for each compound. The Folin–Ciocâlteu phenol reagent, sodium carbonate (Na_2_CO_3_; 99% purity), tannic acid (Ph Eur purity), aluminum chloride hexahydrate (AlCl_3_ × 6 H_2_O; Ph Eur purity), quercetin (98% purity), 3-[4,5-dimethylthiazol-2-yl]-2,5-diphenyl tetrazolium bromide (MTT), doxorubicin (98% purity), and ethanol (EtOH) were provided by Merck (Darmstadt, Germany), while media, cofactors, and antibiotics for cell cultures were from Aurogene (Rome, Italy).

### 2.2. Extraction, Isolation, and Identification of Metabolites of R. tinctoria Lichen Material

*R. tinctoria* was collected in the area between Ostia City and Parco Archeologico of Ostia Antica in July 2022. The lichen *R. tinctoria* has been recognized by the Department of Environmental Biology, Sapienza University of Rome, through comparison of its morphological data with those reported in the literature. A representative sample of this collection is also stored in our laboratory for further reference under voucher number code RT20220715. Then, 15 g of thalli was successively extracted by Soxhlet apparatus with solvents of increased polarity, i.e., *n*-hexane, dichloromethane, and methanol. The resulting separated solutions were concentrated at reduced pressure via Rotavapor at a temperature of 35 °C, 40 °C, and 40° C, respectively, obtaining three extracts named DF1, DF2, and DF3, with a yield of 0.17, 1.3, and 36%, respectively. Another extract, named DF4 (24% yield), was prepared from a united double-consecutive maceration of 10 g of thalli in 150 mL of methanol for 24 h after concentration at reduced pressure via Rotavapor at a temperature of 40 °C. Purification of DF3 and DF4 was performed by CC on silica gel using CH_2_Cl_2_ 100% → CH_2_Cl_2_:MeOH (9:1 *v*/*v*) as a mobile phase. Three main compounds were isolated from DF3 and DF4: erythrin (**1**), methyl orsellinate (**2**), and montagnetol (**3**).

#### Characterization of Compounds **1**–**3**

Erythrin (**1**): Mp 158–160 °C; ^1^H NMR (CD_3_OD, 400 MHz) δ 6.63 (m, 2H), 6.27 (d, *J* = 2.4 Hz, 1H), 6.21 (d, *J* = 2.5 Hz, 1H), 4.64 (m, 1H), 4.45 (dd, *J* = 11.5, 6.6 Hz, 1H), 3.91 (m, 1H), 3.79 (dd, *J* = 13.6, 6.0 Hz, 1H), 3.75–3.64 (m, 2H), 2.56 (s, 6H); IR: ν 3484, 1655, 1608 (cm^−1^); t_R_: 12.44 min. MS: [M − H]^−^ = 421 *m*/*z*, [M + H]^+^ = 423 *m*/*z*, [M + Na]^+^ = 445 *m*/*z*, principal fragments: ES-, CE 17 eV: 167, 149, 271 *m*/*z*; ES+, CE 10 eV: 151 *m*/*z*.

Methyl orsellinate (**2**): Mp 148–150 °C; ^1^H NMR (DMSO-*d_6_*) δ 10.66 (s br, 1H), 9.98 (s br, 1H), 6.16 (m, 2H), 3.79 (s, 3H), 2.28 (s, 3H); IR: ν 3366, 3309, 1613 (cm^−1^); t_R_: 13.33 min. MS: [M − H]^−^ = 181 *m*/*z*, [M + H]^+^ = 183 *m*/*z*, principal fragments: ES-, CE 10 eV: 149 *m*/*z*; ES+, CE 14 eV: 151 *m*/*z*.

Montagnetol (**3**): Mp 136–138 °C; ^1^H NMR (CD_3_OD) δ 6.20 (d, *J* = 1.8 Hz, 1H), 6.15 (d, *J* = 2.3 Hz, 1H), 4.59 (dd, *J* = 11.6, 2.8 Hz, 1H), 4.37 (dd, *J* = 11.6, 6.6 Hz, 1H), 3.92–3.86 (m, 1H), 3.81 (m, 1H), 3.66–3.61 (m, 2H), 2.15 (s, 3H); IR: ν 3394, 3202, 1634 (cm^−1^); t_R_: 6.75 min. MS: [M − H]^−^ = 271 *m*/*z*, [M + H]^+^ = 273 *m*/*z*, [M + Na]^+^ = 295 *m*/*z*, principal fragments: ES-, CE 12 eV: 149, 167 *m*/*z*; ES+, CE 12 eV: 151 *m*/*z*.

### 2.3. HPLC Analysis

High-Performance Liquid Chromatography (HPLC) analysis was carried out with the following instrumentation and parameters: 1260 Infinity II Prime LC System (Agilent, Santa Clara, CA, USA); 1260 Infinity II autosampler; HS 1260 Infinity II diode array detector (Agilent); InfinityLab Poroshell 120 EC-C18 column (3.0 mm × 150 mm, 2.7 µm) (Agilent); solvent A (MilliQ water), solvent B (acetonitrile); gradient program: 5–100% solvent B linearly for 30 min; flow rate 0.5 mL/min; temperature of the column 30 °C; lambda 254 nm.

### 2.4. Analyses by Gas Chromatography–Mass Spectrometry (GC-MS)

#### 2.4.1. SPME Sampling

To identify the volatile chemical profile of the untreated *R. tinctoria*, SPME sampling technique was used. Further, 500 mg of the lichen was inserted in a 7 mL glass vial equipped with a PTFE-coated silicone septum. An SPME device from Supelco (Bellefonte, PA, USA) with 1 cm fiber coated with 50/30 μm DVB/CAR/PDMS (divinylbenzene/carboxen/polydimethylsiloxane) was used to adsorb the volatile compounds. The fiber was initially conditioned at 270 °C for 30 min. After reaching equilibrium, the fiber was exposed to the headspace of the sample for 30 min at 40 °C. Lastly, to desorb the components, the SPME fiber was inserted in the GC injector maintained at 250 °C in spitless mode.

#### 2.4.2. GC-MS Analysis

To perform the analyses of the untreated matrix and of DF1, DF2, and DF3 extracts, a Clarus 500 model Perkin Elmer (Waltham, MA, USA) gas chromatograph equipped with FID (flame detector ionization) and coupled with a single-quadrupole mass spectrometer (Clarus 500 model Perkin Elmer) was used. A capillary column (Varian Factor Four VF-1) was housed in the GC oven, whose programmed temperature was set initially at 60 °C, then at a gradient of 7 °C/minute to 170 °C and a gradient of 8 °C/minute to 250 °C for 25 min. The injector GC was set at 270 °C. Helium was used as carrier gas at a constant rate of 1 mL/min. MS detection was performed with electron ionization (EI) at 70 eV, operating in the full-scan acquisition mode in the *m*/*z* range 40–550 amu. The identification of compounds was performed by the comparison of the MS-fragmentation pattern of the analytes with those of pure components stored in the Wiley 2.2 and Nist 11 mass spectra libraries database. Further, the linear retention indices (LRIs) were calculated using a series of alkane standards (C_8_–C_25_ n-alkanes-Agilent) and then compared with those available in the literature. The relative amounts of the components were expressed as percent peak area relative to total peak area without the use of an internal standard and any factor correction. The analysis was carried out in triplicate.

#### 2.4.3. GC-MS Analysis of the Extracts after Derivatization

To describe the non-volatile content of extracts DF3 and DF4, a derivatization reaction was performed. For this purpose, about 1mg of each extract was added to 300 µL of pyridine and 100 µL of *bis*-(trimethylsilyl) trifluoroacetamide (BSTFA), with heating at 80 °C for 60 min. One μL of the silylated sample was manually injected at 280 °C into the GC injector. The analysis was performed using the same apparatus GC-MS and the same capillary column (Varian Factor Four VF-1) described in the previous section.

The GC oven temperature program was as follows: 80 °C at the beginning; a gradient of 7 °C/min up to 170 °C; a gradient of 8 °C/min up to 250 °C; a gradient of 8 °C/min up to 300 °C for 25 min. The identification of compounds was achieved on the basis of the similarity percentage and comparison of the obtained mass spectra with those reported in software NIST 11 data library.

### 2.5. Spectrophotometric Analysis of Total Polyphenols and Tannins

Total polyphenols and tannin contents were determined according to the Folin–Ciocâlteu method [40]; 20 μL of the tested sample was added to 100 μL of the Folin–Ciocâlteu reagent and 80 μL of a sodium carbonate solution at a concentration of 7.5% *w*/*v*. After incubation for 2 h, the absorbance was measured at 765 nm by an Epoch Microplate Spectrophotometer (BioTek, AHSI, Milan, Italy). Tannins were calculated by the difference between the amount of polyphenols in the extracts and that in their supernatants after precipitation with polyvinylpyrrolidone (100 mg per mL of extract). The total content was determined from the calibration curves of tannic acid (y = 31055∗x + 0.04217) and expressed as tannic acid equivalents (TAEs) per mg of extract.

### 2.6. In Vitro Cytotoxic Activity

#### 2.6.1. Cell Culture

To perform this study, human non-small A549 lung cancer and extrahepatic Mz-ChA-1 cholangiocarcinoma cells, along with the human noncancerous epithelial BEAS-2B bronchial cells and SV40-immortalized H69 cholangiocytes [41], were used. A549 cells were kindly provided by Prof. Lucia Nencioni (Dept. of Public Health and Infectious Diseases, Sapienza University of Rome, Rome, Italy), while Mz-ChA-1 and H69 cells were donated by Prof. Romina Mancinelli (Dept. of Anatomical, Histological, Forensic and Orthopedic Sciences, Sapienza University of Rome, Rome, Italy) and Prof. G. Alpini (Indiana University School of Medicine, Indianapolis, IN, USA). BEAS-2B cells were provided by Sigma-Aldrich (St. Louis, MO, USA). All the cell lines were cultured at a density of approximately 2 × 10^4^ cells/cm^2^ in suitable media and co-factors under standard conditions (37 °C and 5% CO_2_), according to previous studies [42,43]. The growth media were replaced twice per week; subcultures were prepared when the cells achieved approximately 80% confluency.

#### 2.6.2. Cytotoxicity Evaluation

The 3-[4,5-dimethylthiazol-2-yl]-2,5-diphenyl tetrazolium bromide (MTT) assay was used to determine the effect of the treatments on the cell viability, according to previous methods [43]. In brief, 2 × 10^4^ cells/well were cultured into 96-well microplates for 24 h. Then, they were subjected to a treatment with progressive concentrations of the tested extracts for further 24 h. Further, 1% *v*/*v* ethanol (EtOH) was used as negative control, whereas the known cytotoxic agent doxorubicin was used as positive control. The effect of the treatments on the cell viability was expressed as percentage of the vehicle control. A significant level of cytotoxicity is achieved when a decrease in the cell viability exceeding 30% of the control is observed [44].

### 2.7. In Vitro Antifungal Assays

#### 2.7.1. Antifungal Susceptibility Test

*Candida albicans* ATCC 10231 was obtained from the American Type Culture Collection (ATCC, Rockville, MD, USA). The antifungal effects of the isolated compounds and of the extracts against *C. albicans* ATCC 10231 were measured using the broth microdilution method following the standardized methods for yeast [45,46]. *C. albicans* was cultured on Sabouraud dextrose agar (Sigma Aldrich, St. Louis, MO, USA) at 35 °C for 24 h. The final concentration of the inoculum was 2500 cells/mL. The extracts and the compounds were firstly dissolved in DMSO and then diluted at least 100-times with RPMI-1640 medium buffered with MOPS (4-morpholinepropanesulfonic acid). The concentrations ranged from 512 μg/mL to 8 μg/mL for DF3 and DF4, and from 128 μg/mL to 1 μg/mL for DF3C1, DF3C1, and DF4C1. The minimal inhibitory concentration (MIC) was calculated by comparison of the growth in each well with that of the drug-free control after 24 h. Each experiment was conducted in triplicate and replicated at least three times on different dates.

#### 2.7.2. Anti-Adhesion Activity

Presterilized, polystyrene, flat-bottom 48-well microtiter plates were used to investigate anti-*Candida* adhesion property of the extracts and the compounds [47,48]. For this, 200 μL aliquot of 1.0 × 10^6^ cell/mL *C. albicans* suspension was transferred into microtiter plate wells together with the extract solutions at concentrations from 512 μg/mL to 32 μg/mL or of the compound solutions at concentrations from 128 μg/mL to 8 μg/mL, and the plate was incubated for 90 min and 24 h at 37 °C [47]. After that time, the cell suspensions were aspirated, and each well was washed twice with PBS to remove loosely adherent cells. Thus, CV assay was used to quantitatively study *Candida* adhesion (90 min) and early biofilm formation (24 h) [49]. The biomass was measured at 590 nm using a microplate reader.

#### 2.7.3. Antibiofilm Formation Activity

*Candida* biofilm was established on flat-bottomed, 48-well microtiter plates, following previous protocols with minor modifications [50]. A suspension of 1.0 × 10^5^ cells/mL of *C. albicans* was prepared in RPMI-1640 buffered with MOPS (4-morpholinepropanesulfonic acid) and added to each well together with 200 μL of the extract solutions at concentrations from 512 μg/mL to 32 μg/mL or of the compound solution at concentrations from 128 μg/mL to 8 μg/mL. Plates were then incubated for 48 h at 37 °C. After biofilm formation, the medium was aspirated, and non-adherent cells were removed by washing twice with phosphate-buffered saline (PBS). *C. albicans* biomass was quantified using CV assay [49].

#### 2.7.4. Reduction in Preformed Biofilm

*Candida* biofilm was formed as previously described [50]. For example, 200 μL of 1.0 × 10^7^ cells/mL suspension of *C. albicans* was added to polystyrene flat-bottomed, 48-well microtiter plates and incubated for 24 h. After this interval, the medium was aspirated, and non-adherent cells were removed by washing twice with phosphate-buffered saline (PBS). Then, 200 μL of the extract solutions at concentrations from 512 μg/mL to 32 μg/mL or of the compound solutions at concentrations from 128 μg/mL to 8 μg/mL was added on the preformed biofilm and plates incubated for additional 24 h. After removing the media, wells were washed again with PBS. Biomass of preformed biofilm was estimated through CV assay [49]. Furthermore, the experiments were replicated, and the assessment of the metabolic activity of preformed biofilms was quantified using the XTT [2,3-*bis*(2-methoxy-4-nitro-5-sulfophenyl)-2*H*-tetrazolium-5-carboxanilide] reduction assay [51]. The experiment was performed three times independently in triplicate, and the results were expressed as mean ± standard deviation (SD).

### 2.8. In Vivo Antifungal Assay

#### 2.8.1. Galleria Mellonella Survival Assay

The survival assay was carried out as described by Cairone and colleagues [52]. *G. mellonella* larvae weighed 0.3 ± 0.03 g and were obtained from Todaro Sport (Rome, Italy). Nine groups of larvae each containing 10 larvae were inoculated in the last left proleg with *C. albicans* suspension and 10 μL of compounds **1**–**2** at concentrations of 512 μg/mL, 256 μg/mL, and 128 μg/mL, or DF3 at concentrations of 2048 μg/mL, 1024 μg/mL, and 512 μg/mL. Three control groups were used, one with no treatment applied, one pierced and inoculated with sterile saline solution, and one treated with *C. albicans* suspension. The larvae were subsequently placed in an incubator set at 37 °C and observed for 120 h. They were considered to be deceased when they showed no response to gentle pressure applied with forceps. Each trial was replicated three times, and the outcomes were documented as the survival percentage.

#### 2.8.2. In Vivo Toxicity Studies

The toxicity of compounds **1** and **2** and extract DF3 was investigated in vivo using *G. mellonella* larvae. *G. mellonella* larvae were the same as reported in Section 2.8.1. Three groups of ten larvae were pierced in the last left proleg with 10 μL of the compounds (concentrations of 512 μg/mL, 256 μg/mL, and 128 μg/mL) or of the extract (concentrations of 2048 μg/mL, 1024 μg/mL, and 512 μg/mL). Three control groups (each consisting of 10 larvae) were established: one group underwent no treatment; one group was treated with sterile saline solution; one group was treated with *C. albicans* suspension. The larvae were subsequently incubated at 37 °C and observed for 120 h, with death determined as reported in Section 2.8.1. Each experiment was replicated three times, and the results were reported as the percentage of survival.

### 2.9. Statistical Analysis

Results are expressed as the mean ± standard error (SE) of at least three experiments each with three technical replicates. GraphPad Prism™ (Version 6.00) software (GraphPad Software, Inc., San Diego, CA, USA) was used to perform the statistical analysis and graphical representation of data. The one-way analysis of variance (one-way ANOVA), followed by Dunnett’s multiple comparison post test, was applied to assess statistically significant differences (*p* value < 0.05) among multiple treatments, the Student’s *t*-test for comparison of data between two groups, and the extra sum-of-square F test for evaluating if the IC_50_ values differ for each dataset.

## 3. Results and Discussion

### 3.1. Extraction and Spectrophotometric Analysis of Total Polyphenols and Tannins

*R. tinctoria* was successively extracted using Soxhlet apparatus with solvents of increased polarity, i.e., *n*-hexane, dichloromethane, and methanol, giving extracts named DF1, DF2, and DF3, respectively. Extraction with both apolar solvents resulted in a lower yield (0.17–1.3%), while a higher amount of extract was obtained with methanol (36%). In addition, a methanolic extract (DF4) of *R. tinctoria* was obtained by maceration; as expected, the yield was lower (24%) than DF3 because of a lower temperature of extraction. The spectrophotometric analysis of the methanolic extracts (DF3 and DF4) highlighted the presence of total polyphenols and tannins in both the tested extracts, especially in DF3; in both samples, tannins accounted for approximately two-thirds of the total polyphenols (Table 1). Our results agree with studies highlighting that phenolic compounds are the most relevant secondary metabolites in lichenized fungi [35,53]. For instance, Mendili et al. [54] showed that the *Flavoparmelia caperata* (L.) Hale and *Squamarina cartilaginea* (With.) P. James lichens were richest in total phenolic and proanthocyaninds, respectively. Similarly, phenolic compounds were detected in the *Ramalina pacifica* Asahina as well as in *R. montagnei* and *Roccella phycopsis* Ach. [55,56].

### 3.2. Isolation, Characterization, and Quantification of Erythrin (***1***), Methyl Orsellinate (***2***), and Montagnetol (***3***)

**Figure 1:**
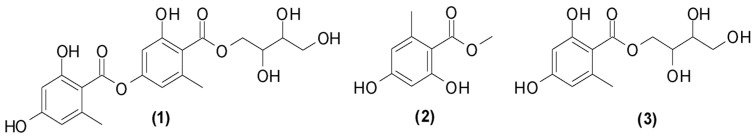
Non-volatile compounds isolated from *R. tinctoria* methanolic extracts.

The fractionation of DF3 and DF4 by column chromatography on silica gel gave three pure compounds that were identified as erythrin (**1**), methyl orsellinate (**2**), and montagnetol (**3**) through Fourier-transform infrared spectroscopy (FTIR), mass spectrometry (MS), and melting point (MP) analysis as well as a comparison with proton nuclear magnetic resonance (^1^H-NMR) data in the literature (Figure 1) [57,58].

The presence of these compounds has previously been reported in the species [12,13,14,15]. The quantification of **1**–**3** in DF3 and DF4 was carried out by HPLC-DAD. As shown in Table 2, erythrin is the main component in DF4, while methyl orsellinate is the main one in DF3.

### 3.3. SPME-GC-MS Chemical Composition

To describe the chemical profile of untreated *R. tinctoria*, the matrix was analyzed via SPME-GC-MS. In total, 14 components belonging to different chemical classes were identified (Table 3). The main classes were terpenoids, aliphatic aldehydes, and carbonyl compounds. The main molecule was the hydrocarbon 8-heptadecene (73.5%). The three detected and identified terpenoids were *β*-cyclocitral, *trans*-*β*-ionone, and sandaracopimaradiene. Two furan derivatives were also found, namely 2-pentylfuran and 2(4*H*)-benzofuranone,5,6,7,7*a*-tetrahydro-4,4,7*a*-trimethyl. The present work is the first to describe the volatile chemical components of untreated *R. tinctoria* via the SPME-GC/MS technique.

### 3.4. Chemical Composition of the Volatile Components of the Extracts

The GC analyses carried out on the extracts allowed us to detect the volatile compounds present in these samples (Table 4). DF1 was qualitatively the richest extract, with seven identified components; DF3 was characterized by only two components, whereas DF2 and DF4 by only one. In detail, in DF1 palmitic acid (44.2%) and 2,5-furandione, dihydro-3-tatradecyl (35.3%) was the most abundant, followed by 2(4H)-benzofuranone and 5,6,7,7a-tetrahydro-4,4,7a-trimethyl (11.5%). In DF2 and DF4, 2,5-furandione,dihydro-3-tatradecyl and orcinol were found, respectively. On the other side, in DF3, methyl orsellinate (51.0%) and 2,5-furandione,dihydro-3-tatradecyl (49.0%) were detected.

### 3.5. Chemical Composition of Extracts DF3 and DF4 after Derivatization

Direct injection analyses of the silylated extracts allowed for the identification of only erythritol in DF3 and DF4 extracts, as reported in Table 5.

### 3.6. In Vitro Cytotoxicity Assay

The antiproliferative activity of DF3 and DF4 extracts (concentration range from 10 to 1000 µg/mL; 1:1.3 to 1:5 dilution factor) and **1**–**3** (concentration range from 10 to 500 µg/mL; 1:1.3 to 1:5 dilution factor) was assayed in terms of viability inhibition in human A549 lung cancer and Mz-ChA-1 cholangiocarcinoma cells. In addition, tolerability studies in human epithelial BEAS-2B bronchial cells and H69 cholangiocytes were performed in order to establish the possible selective cytotoxicity of the tested samples to cancer cells.

**Figure 2:**
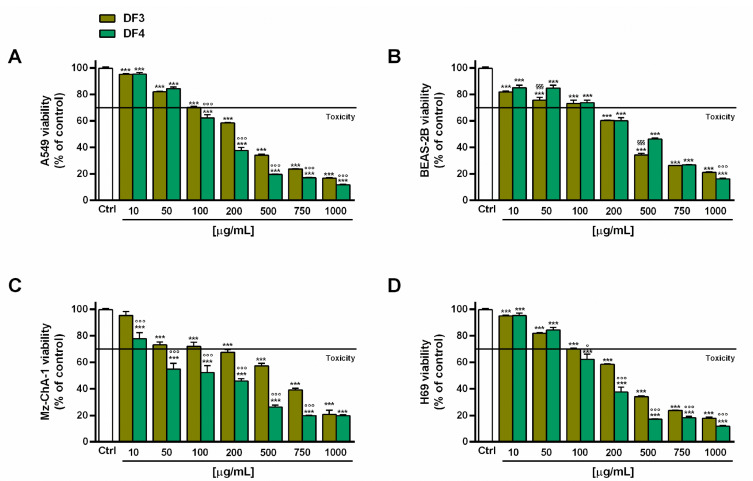
Effect of DF3 and DF4 extracts on the viability of the the A549 lung cancer cells (**A**), noncancerous epithelial bronchial BEAS-2B cells (**B**), Mz-ChA-1 cholangiocarcinoma cells (**C**) and H69 cholangiocytes (**D**). Data represent the average and standard error of at least three independent experiments, each one with at least three technical replicates (*n* = 9). *** *p* < 0.001, significantly different with respect to control (ANOVA followed by Dunnett’s multiple comparison post test). ° *p* < 0.05 and °°° *p* < 0.001, significantly lower than DF3 (Student’s *t*-test). ^§§§^ *p* < 0.001, significantly lower than DF4 (Student’s *t*-test).

DF3 and DF4 extracts induced progressive cytotoxic effects, starting from concentrations of 100 and 50 µg/mL, respectively (Figure 2A,C), in both A549 and Mz-ChA-1 cells. DF4 was found to be more potent than DF3; in fact, at a concentration of 100 µg/mL, it induced a 40% and almost 50% inhibition in A549 and Mz-ChA-1 cells, respectively, despite around 30% cell viability inhibition by DF3. This evidence was further confirmed by the IC_50_ values of DF3, which were about 1.5- and 4-fold higher than those of DF4 in A549 and Mz-ChA-1 cells, respectively (Table 6).

In the noncancerous BEAS-2B cells, the extracts exhibited a similar toxicity profile, as also confirmed by the IC_50_ values. In particular, they were nontoxic up to 100 µg/mL, with early toxicity signs (about 40% cytotoxicity) at a concentration of 200 µg/mL (Figure 2B). Conversely, DF3 appeared to be slightly less toxic than DF4 in H69 cholangiocytes; indeed, at 100 µg/mL, it induced about 30% inhibition of cell viability, despite 40% inhibition by DF4 (Figure 2D). The IC_50_ value of DF3 was 1.6-fold higher than that of DF4 (Table 6). Interestingly, DF4 exhibited a toxicity profile about 2-fold lower than that highlighted in cancerous cells (Table 6).

**Figure 3:**
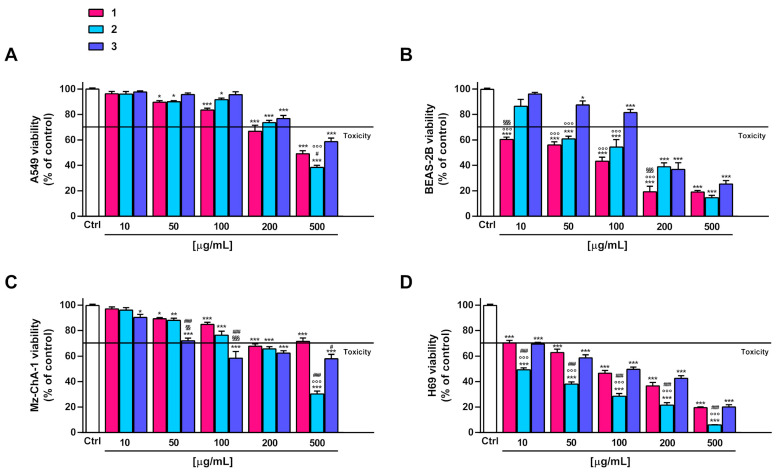
Effect of **1**–**3** on the viability of the A549 lung cancer cells (**A**), noncancerous epithelial bronchial BEAS-2B cells (**B**), Mz-ChA-1 cholangiocarcinoma cells (**C**) and H69 cholangiocytes (**D**). Data represent the average and standard error of at least three independent experiments, each one with at least three technical replicates (*n* = 9). * *p* < 0.05, ** *p* < 0.01 and *** *p* < 0.001, significantly different with respect to control (ANOVA followed by Dunnett’s multiple comparison post test). ^#^ *p* < 0.05 and ^###^ *p* < 0.001, significantly lower than **1** (Student’s *t*-test). ^§§^ *p* < 0.01 and ^§§§^ *p* < 0.001, significantly lower than **2** (Student’s *t*-test). °°° *p* < 0.001, significantly lower than **3** (Student’s *t*-test).

Under our experimental conditions, compounds **2** and **3** exhibited weak in vitro anticancer properties (lower than 50% cytotoxicity at the highest concentration tested) in A549 and Mz-ChA-1 cells. Compound **1** was slightly more potent than **2** and **3,** producing at least 60% inhibition at the highest concentration of 500 µg/mL (Figure 3A,B). For all compounds, the IC_50_ values were not evaluable since the maximum cytotoxicity was lower than 80% (Table 6). The compounds, especially **1** and **2**, were markedly toxic in noncancerous BEAS-2B and H69 cells, respectively. Conversely, **3** showed cytotoxic effects starting from a concentration of 200 µg/mL in BEAS-2B cells and from a concentration of 50 µg/mL in H69 cholangiocytes (Figure 3B,D). In fact, at a concentration of 50 µg/mL, **1** and **2** induced at least 40% inhibition of BEAS-2B cell viability, whereas **3** induced lower inhibition (less than 20%). At the same concentration, **2** lowered the H69 cell viability by 60%, while **1** and **3** by about 40% (Figure 3B,D).

Although several lichen-derived compounds have been assessed as potential anticancer agents [19,20,22], the investigations into isolated compounds **1**–**3** were limited or lacking. Huy and Bui [59] isolated compounds **1** and **3** from the lichen *Roccella sinensis* and showed they were nontoxic in MCF-7 (breast cancer cell line), HeLa (cervical cancer cell line), HepG2 (liver hepatocellular carcinoma cell line), and NCI-H460 (human lung cancer cell line) at a concentration of 100 µg/mL. Accordingly, these substances were nontoxic or low-toxic at the same concentration in A549 and Mz-ChA-1 cells under our experimental conditions. Conversely, Roser et al. [60] reported that lecanoric acid, which is a structural analogue of compound **1**, affected the cell viability and colony formation, and it induced a cell cycle block in HCT-116 colon cancer cells, despite exhibiting lower cytotoxic effects in noncancerous primary human immune and endothelial cells. Under our experimental conditions, the pure compounds **1**, **2**, and **3** exhibited higher toxicity in noncancerous cells (specifically, **1** > **2** > **3** in BEAS-2B cells and **2** > **1** > **3** in H69 cells) compared to cancerous ones, despite an opposite trend for the extracts. This suggests that the harmful effects of the pure compounds may be mitigated within the phytocomplex by the presence of protective or antagonistic agents. Further studies are needed to elucidate the contribution of **1**–**3** to the in vitro anticancer activity of DF3 and DF4 extracts from *R. tinctora* lichen and their tolerability in vivo.

### 3.7. In Vitro Antifungal Activity

#### 3.7.1. Antifungal Susceptibility Test

The antifungal activity of extracts and isolated compounds against *C. albicans* planktonic cells was examined following the standardized broth microdilution method for yeasts (CLSI M27-A3) [45,46]. The results presented in Table 7 showed that compound **2** inhibited *C. albicans* planktonic cell growth by 50% at a concentration of 87 μg/mL. Indeed, compounds **1** and **3** and DF4 and DF3 extracts showed no activity against the planktonic growth of *C. albicans* at the highest concentration (512 μg/mL)

The reported results in Table 7 are perfectly in accordance with previous studies on the same topic. In fact, erythrin (**1**) has not shown any antifungal activity against different fungal strains including *C. albicans* [61]. Indeed, compound **2** has already shown high antifungal effects against *Alternaria solani* and *Fusarium oxysporum,* with inhibition percentages of 58.41% and 67.39% at a concentration of 100 µg/mL [62]. Lastly, montagnetol (**3**) has shown very poor effects against *C. albicans,* with an inhibition zone diameter of 0.125 mm [33]. From the literature survey, it was possible to observe that the absence of the alcoholic chain seems to favor the activity as well as the presence of only one aromatic group. Yet, this hypothesis needs more in-depth pharmacological studies to be fully verified.

#### 3.7.2. Anti-Candida Adhesion Activity

Given the importance of *C. albicans* adhesion to abiotic surfaces in the establishment of hard-to-treat infections, the following investigation focused on the ability of the extracts to inhibit this virulence factor [50,63]. An anti-adhesion assay was conducted following the method previously described by Bandara and colleagues [47] on polystyrene flat-bottomed plates. The total biomass of the biofilm was evaluated using a crystal violet (CV) assay, and the results were reported as the percentage of inhibition.

**Figure 4:**
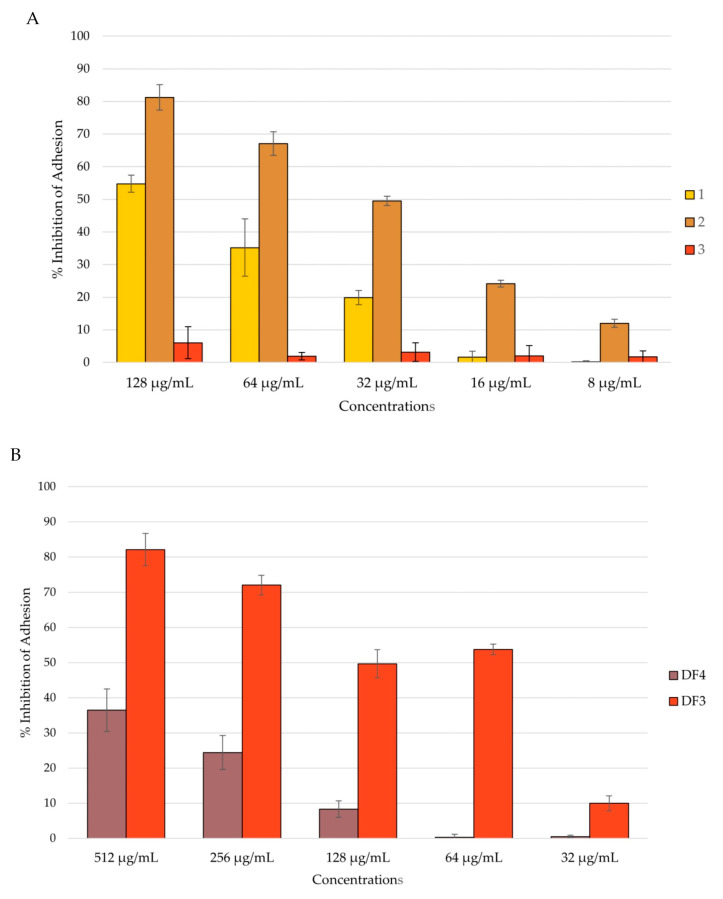
Percent rate of inhibition of *C. albicans* adhesion after 90 min of incubation: (**A**) by compounds **1**–**3**; (**B**) by DF4 and DF3. The results are expressed as mean ± standard deviation (SD) of three different experiments conducted on different dates.

Among the isolated compounds, **2** showed the highest percentage of inhibition of *C. albicans* adhesion, with a reduction of 81% at a concentration of 128 μg/mL. Compound **1** inhibited *C. albicans* adhesion by 55% at a concentration of 128 μg/mL. Compound **3** had no activity against *C. albicans* adhesion (Figure 4A). Among the extracts, DF3 showed the best activity, with around a 50% reduction in adhesion at a concentration of 64 μg/mL (Figure 4B).

#### 3.7.3. Anti-Candida Biofilm Formation

**Figure 5:**
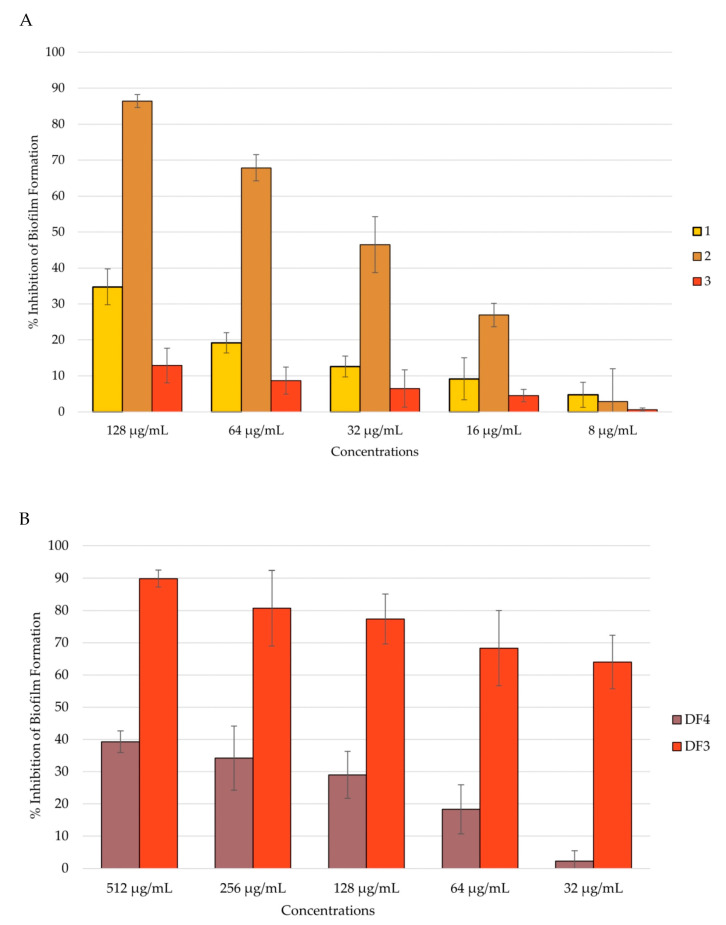
Percent rate of inhibition of *C. albicans* biofilm after 24 h of incubation: (**A**) by **1**–**3**; (**B**) by DF4 and DF3. The results are expressed as mean ± standard deviation (SD) of three different experiments conducted on different dates.

*Candida* adhesion is followed by the colonization and formation of the biofilm, which is a community of cells with the ability to secrete the extracellular matrix (ECM). Biofilm-characterized infections are the most difficult to treat, and preventing biofilm formation could be of major importance. An anti-*Candida* biofilm formation assay was conducted after 24 h and 48 h of biofilm incubation, and the total biomass was evaluated using the CV assay [49,64]. All tested extracts showed the ability to inhibit *C. albicans* biofilm formation in a dose-dependent manner after 24 h of incubation. Even in this case, compound **2** and extract DF3 were shown to be the most active. In fact, compound **2** inhibited the biofilm formation by 86% at a concentration of 128 μg/mL (Figure 5A), and DF3 showed a percent rate of inhibition of nearly 90% at a concentration of 512 μg/mL (Figure 5B). Further, **1** and DF4 had a percent rate of inhibition lower than 40%, while **3** was confirmed to have no activity against *C. albicans* mean virulence factors, such as adhesion and biofilm formation (Figure 5A).

**Figure 6:**
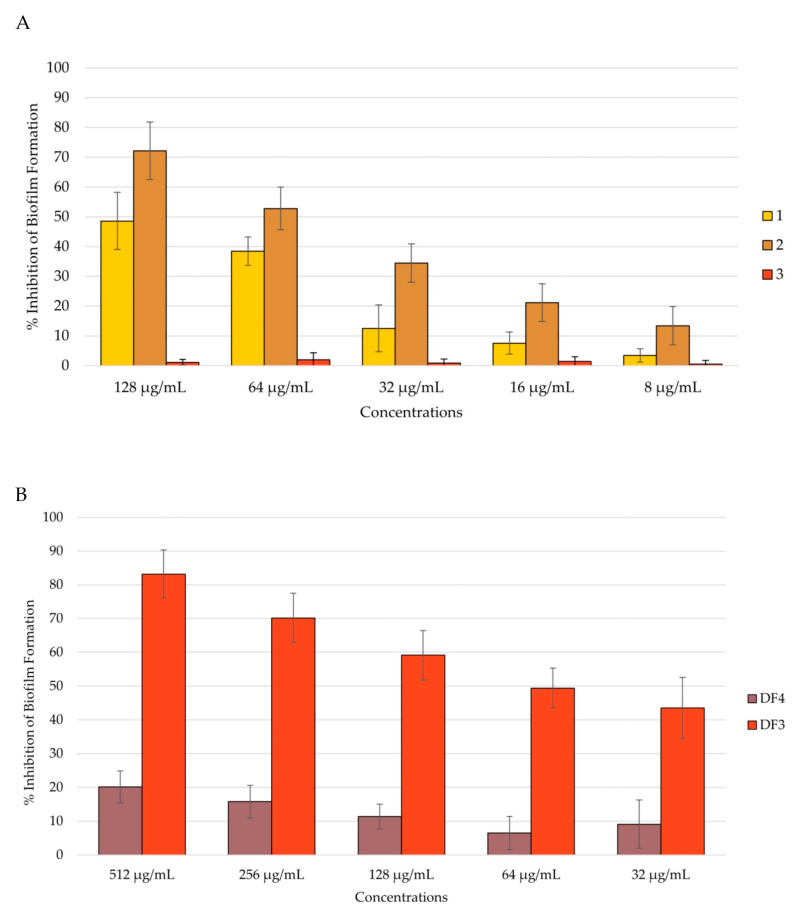
Percent rate of inhibition of *C. albicans* formation after 48 h of incubation: (**A**) by **1**–**3**; (**B**) by DF4 and DF3. The results are expressed as mean ± standard deviation (SD) of three different experiments conducted on different dates.

After 48 h of incubation, **2**’s anti-biofilm formation activity decreased to 72% at 512 μg/mL (Figure 6A), remaining the most active together with DF3, which inhibited biofilm formation by 83% after 48 h at 512 μg/mL (Figure 6B). Compound **1** inhibited biofilm formation by 49% after 48 h at 128 μg/mL, while **3** remained inactive (Figure 6B). DF4 anti-biofilm formation considerably decreased at 48 h, with respect to the result obtained at 24 h (percent rate of 20% at 512 μg/mL). All tested extracts showed dose-dependent capacity to inhibit biofilm formation, even after 48 h of incubation (Figure 6B).

#### 3.7.4. Anti-Candida Mature Biofilm Inhibition

**Figure 7:**
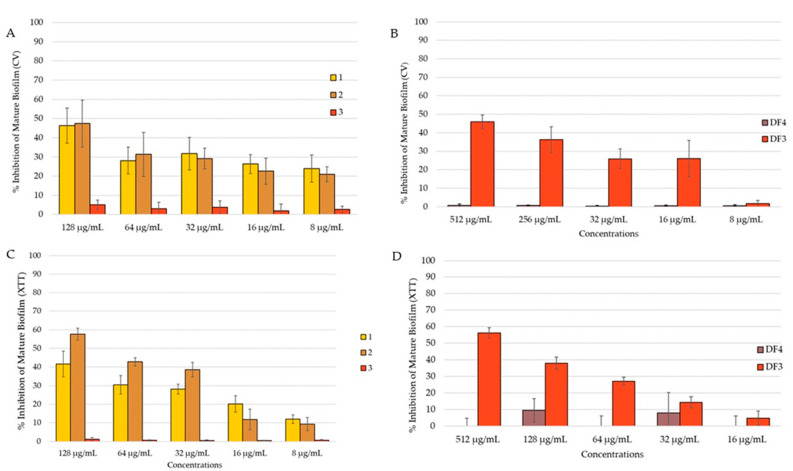
Percent rate of reduction in *C. albicans* mature biofilm: (**A**) by **1**–**3** obtained using CV assay; (**B**) by DF4 and DF3 obtained using CV assay; (**C**) by 1–3 obtained using XTT assay; (**D**) by DF4 and DF3 obtained using XTT assay. The results are expressed as mean ± standard deviation (SD) of three different experiments conducted on different dates. Percent rate of reduction in *C. albicans* mature biofilm obtained using XTT assay. The results are expressed as mean ± standard deviation (SD) of three different experiments conducted on different dates.

The *C. albicans* mature biofilm was grown for 24 h on polystyrene flat-bottomed plates, before adding the extract solutions at different concentrations and incubated for an additional 24 h. A reduction in the mature biofilm was investigated using both CV and XTT assays, with the aim to evaluate the total biomass and the metabolic activity, respectively. Due to the high resistance of the biofilm, the activity of the extracts decreased against the preformed biofilm. Compound **2** reduced the mature biofilm by 47% in the CV assay (Figure 7A) and by 58% in the XTT assay (Figure 7C) at 128 μg/mL. At the same concentration, **1** reduced the mature biofilm by 46% (Figure 7A). DF3 showed percent rate of inhibition of 46% and 56%, respectively, in the quantification of the biofilm (CV assay, Figure 7B) and in the evaluation of biofilm metabolic activity (XTT assay, Figure 7D) at a concentration of 512 μg/mL. Compound **3** and DF4 had no activity against the C. *albicans* mature biofilm.

### 3.8. In Vivo Antifungal Assays

**Figure 8:**
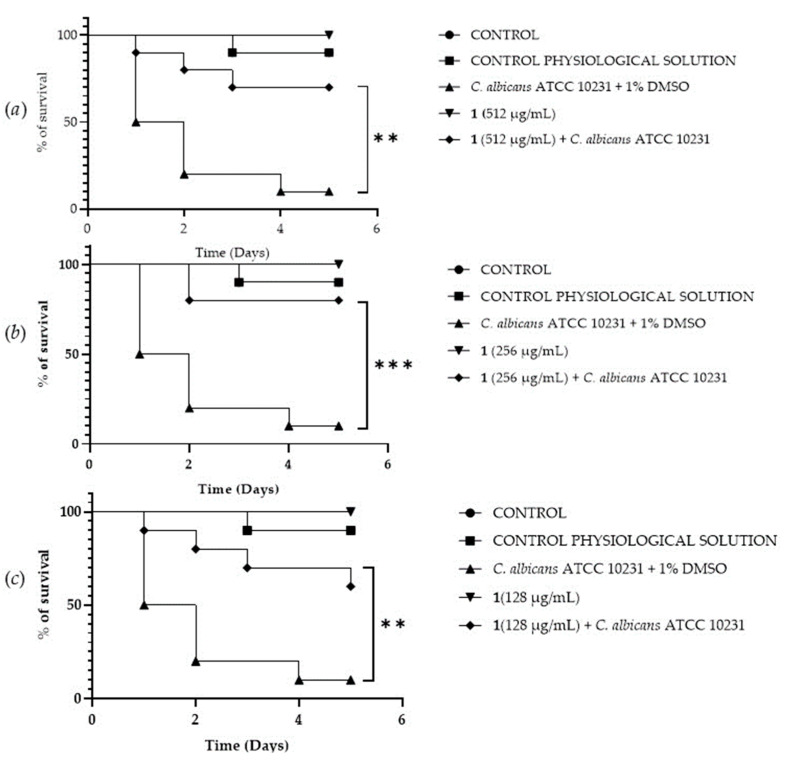
Survival curves of *G. mellonella* larvae infected with *C. albicans* and treated with 512 µg/mL (**a**), 256 µg/mL (**b**), 128 µg/mL (**c**) of **1**. The Mantel–Cox log-rank test was employed to assess the statistical significance with respect to the control. *** indicates *p* < 0.001 compared to the control; ** indicates *p* < 0.01 compared to the control.

**Figure 9:**
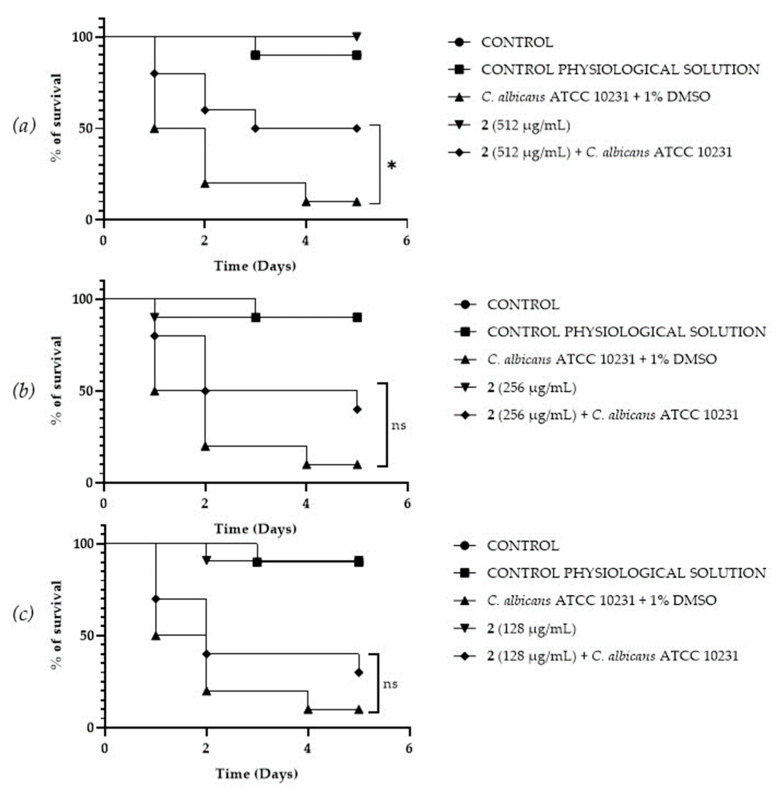
Survival curves of *G. mellonella* larvae infected with *C. albicans* and treated with different concentrations (512 µg/mL (**a**), 256 µg/mL (**b**), 128 µg/mL (**c**)) of **2**. The Mantel–Cox log-rank test was utilized to determine the statistical significance with respect to the control. * denotes *p* < 0.05 compared to the control; ns indicates not significant.

**Figure 10:**
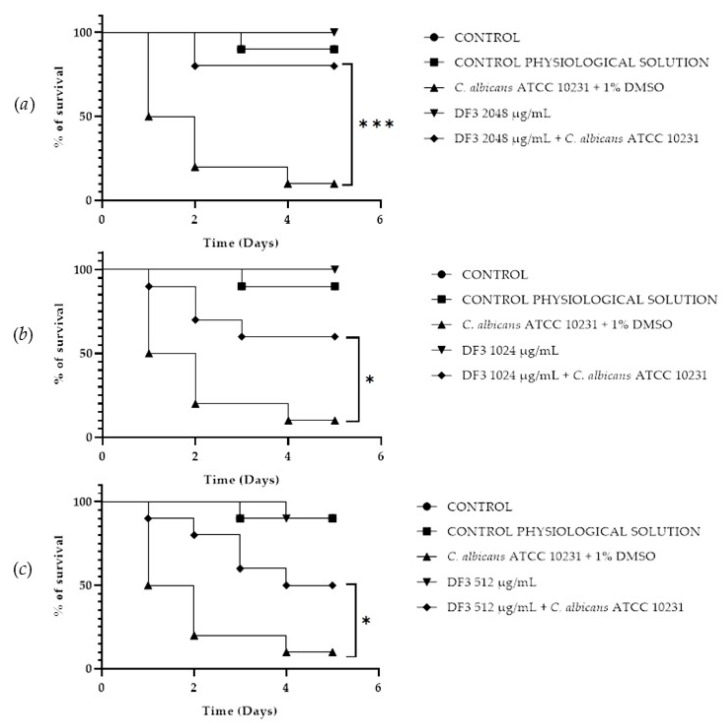
Survival curves of *G. mellonella* larvae infected with *C. albicans* and subjected to treatment with 2048 µg/mL (**a**), 1024 µg/mL (**b**), and 512 µg/mL (**c**) of **DF3**. The Mantel–Cox log-rank test was employed to assess the statistical significance with respect to the control. *** indicates *p* < 0.001 compared to the control; * indicates *p* < 0.05 compared to the control.

A *Galleria mellonella* survival assay was conducted to investigate the antifungal activity and toxicity of **1**, **2** and DF3. *G. mellonella* larvae as an in vivo model were chosen due to the similarities between their immunity system and the mammalian one. Furthermore, *G. mellonella* larvae provide a well-established in vivo model known for its ease of handling and utilization. They are extensively employed to assess the antimicrobial efficacy of both chemical and natural compounds, aligning with the ethical guidelines of the 3Rs (Replacement, Reduction, and Refinement) in animal research [50]. Our findings suggest the ability of the extract and compounds to significantly increase the survival of *G. mellonella* larvae infected with *C. albicans* ATCC 10231. When treated with 256 μg/mL of **1**, the infected larvae mortality rate was significantly (*p* value < 0.001) lower (20%) when compared with the control group mortality rate (90%) (Figure 8b). Compound **2** showed a significant result (*p* value < 0.05) only at 512 μg/mL, increasing the larvae survival rate up to 50% after 5 days, with respect to the infected control survival rate (10%) (Figure 9a). DF3 was found to be significantly active at all three tested concentrations, 2048 μg/mL (*p* value < 0.001), 1024 μg/mL (*p* value < 0.05), and 512 μg/mL (*p* value < 0.05), with respect to infected larvae control (Figure 10). At a concentration of 2048 μg/mL, DF3 decreased the mortality rate to 20%, with respect to the 90% mortality rate of the control. The concentrations of 1024 μg/mL and 512 μg/mL of DF3 decreased mortality to 60% and 70% with respect to the control. The literature data indicate that effective quantities in larvae are comparable to doses applicable in humans [65]. Therefore, considering the highest dose used in the larvae (2048 µg/mL), one might hypothesize that 3.4 g of DF3 should be administered to a 50 kg individual, while for the lowest effective extract dose (512 µg/mL), the amount to be administered to a 50 kg individual should be 0.850 g.

## 4. Conclusions

The phytochemical analysis performed for the first time on different extracts of *R. tinctoria* led to the identification of several compounds, some of which are new for the species. In addition, the biological tests on some of these extracts and their components led to the discovery of new activities associated with them, with good efficacy values. Future research must surely focus on the investigation of the mechanisms of action of the active compounds and extracts for each biological assay reported in this work as well as on testing directly this species, their different extracts, and their singular components for other possible biological assays, given the relatively scarce data in the literature in this context.

## Figures and Tables

**Table 1:** Amounts of total polyphenols and tannins in DF3 and DF4 extracts from *R. tinctoria*, determined as tannic acid equivalents (TAEs). Data are expressed as mean ± standard error (SE) of at least two experiments, each one with at least three technical replicates (*n* = 6).

Extracts	Polyphenols	Tannins
	mg TAE/g Extract	
DF3	43.48 ± 1.04 ***	30.78 ± 1.33 ***
DF4	35.47 ± 0.88	23.22 ± 1.09

*** *p* < 0.001, significant difference in the amounts determined in DF3 with respect to DF4 (*t*-Student Test).

**Table 2:** Amounts of **1**–**3** in DF3 and DF4 extracts from *R. tinctoria*. Data are expressed in mg of compounds/100 mg extract.

Compound	DF3	DF4
**1**	6.90 ± 0.33	22.0 ± 0.66
**2**	23.01 ± 1.10	0.62 ± 0.02
**3**	6.35 ± 0.19	1.80 ± 0.05

**Table 3:** Chemical composition (percentages mean values ± standard deviation) of the volatile compounds of *R. tinctoria*.

Compound ^1^	LRI ^2^	LRI ^3^	*R. tinctoria*
hexanal	804	806	1.9 ± 0.03
heptanal	898	890	0.5 ± 0.02
2-pentylfuran	973	976	1.0 ± 0.01
octanal	997	995	1.1 ± 0.03
nonanal	1104	1100	2.9 ± 0.03
decanal	1184	1179	1.8 ± 0.02
β-cyclocitral	1196	1191	0.5 ± 0.02
pentanal, 2,3-dimethyl-	*	1265	1.4 ± 0.03
*trans*-β-ionone	1470	1475	2.3 ± 0.03
2(4*H*)-benzofuranone, 5,6,7,7*a*-tetrahydro-4,4,7*a*-trimethyl	1525	1531	1.1 ± 0.03
8-heptadecene	*	1678	73.5 ± 1.12
3-pentadecanone	*	1680	3.7 ± 0.04
sandaracopimaradiene	1967	1971	2.7 ± 0.02
2-nonadecanone	2106	2111	5.5 ± 0.06
SUM			99.9
Terpenoids			5.5
Aliphatic aldehydes			9.6
Carbonyl compounds			9.2
Others			2.1

^1^ Components reported in their elution order on non-polar column; ^2^ linear retention indices measured on non-polar column; ^3^ linear retention indices derived from literature; * linear retention indices not available.

**Table 4:** Chemical composition (percentages mean values ± standard deviation) of *R. tinctoria* extracts.

Compound ^1^	LRI ^2^	LRI ^3^	DF1	DF2	DF3	DF4
pentanone	684	681	0.4 ± 0.02	-	-	-
hexanal	804	800	6.2 ± 0.05	-	-	-
orcinol	1369	1378	-	-	-	100.0
6-methyl-6-(5-methylfuran-2-yl)-heptan-2-one	*	1431	0.7 ± 0.03	-	-	-
2(4*H*)-benzofuranone, 5,6,7,7*a*-tetrahydro-4,4,7*a*-trimethyl	1525	1531	11.5 ± 0.06	-	-	-
undecanoic acid	1490	1495	1.6 ± 0.03	-	-	-
methyl orsellinate	*	1666	-	-	51.0 ± 1.10	-
palmitic acid	1973	1981	44.2 ± 0.11	-	-	-
2,5-furandione, dihydro-3-tatradecyl	2350	2355	35.3 ± 0.15	100.0	49.0 ± 1.08	-
SUM			99.9	100.0	100.0	
Phenols			-	-	51.0	100.0
Aliphatic aldehyde			6.2	-	-	
Carbonyl compound			0.4	-	-	
Fatty acids			45.8	-	-	
Others			47.5	100.0	49.0	

^1^ Components reported in their elution order on non-polar column; ^2^ linear retention indices measured on non-polar column; ^3^ linear retention indices derived from literature; * LRI not available; *-* Not detected.

**Table 5:** Chemical composition (percentages mean values) of *R. tinctoria* DF3 and DF4 extracts.

N°	Compounds ^1^	DF3	DF4
1	erythritol	100.0	100.0

^1^ Percentage values of the components of DF3 and DF4 extracts after derivatization.

**Table 6:** Half-maximal inhibitory concentration (IC_50_) values of the cytotoxic effect of DF3 and DF4 extracts, **1**–**3** and the positive control doxorubicin in A549 lung cancer and Mz-ChA-1 cholangiocarcinoma cells, in noncancerous epithelial bronchial BEAS-2B cells and in H69 cholangiocytes. Data represent the mean ± SE of at least three independent experiments with at least three technical replicates (*n* = 9).

Tested Sample	IC_50_ [µg/mL] (CL)			
	A549	BEAS-2B	Mz-ChA-1	H69
DF3	246.9 (219.0–278.3)	260.0 (223.0–303.0)	416.4 (346.0–662.9)	248.5 (223.3–276.6)
DF4	155.5 (129.2–187.1) ***	317.5 (242.2–416.2)	94.6 (70.2–127.4) ***	153.9 (125.5–188.6) ***
**1**	ne ^1^	ne ^2^	ne ^1^	138.0 (78.0–244.1) °
**2**	ne ^1^	102.7 (69.4–150.1) °	ne ^1^	ne ^2^
**3**	ne ^1^	176.7 (121–257.3)	ne ^1^	170.8 (34.3–851.3)
Doxorubicin	17.3 (11.4–42.1)	4.1 (2.3–8.9)	24.5 (13.3–32.9)	15.7 (7.8–29.1)

CL, confidence limits. ne ^1^, not evaluable since the highest inhibitory effect was lower than 80%. ne ^2^, not evaluable being the lower inhibitory effect higher than 40%. *** *p* < 0.001, significantly lower than DF3 (extra sum-of-square F test). ° *p* < 0.05, significantly lower than **3** (extra sum-of-square F test).

**Table 7:** Antifungal activity of **1**–**3**, DF4, and DF3 against *C. albicans* planktonic cells.

	GM MIC_50_ (µg/mL)	GM MIC_90_ (µg/mL)	GM MIC_100_ (µg/mL)
1	>128	>128	>128
2	87	>128	>128
3	>128	>128	>128
DF3	>512	>512	>512
DF4	>512	>512	>512

MIC_50_ = minimum inhibitory concentration of 50% fungal growth; MIC_90_ = minimum inhibitory concentration of 90% fungal growth; MIC_100_ = minimum inhibitory concentration of 100% fungal growth; GM = geometric mean.

## Data Availability

Data are contained within the article.
